# Umbilical vein recanalization with a diameter greater than 6.48mm may promote deterioration of liver reserve function: A retrospective cohort study

**DOI:** 10.1097/MD.0000000000050382

**Published:** 2026-09-04

**Authors:** Feng Ding, Yuan Yang, Jing Wang, Liping Chen

**Affiliations:** a Department of Gastroenterology, Zhongnan Hospital of Wuhan University, Wuhan, Hubei, China.

**Keywords:** liver cirrhosis, portal hypertension, portal pressure gradient, transjugular intrahepatic portosystemic shunt, umbilical vein recanalization

## Abstract

This study aimed to investigate the clinical characteristics of cirrhotic patients with umbilical vein recanalization (UVR) and explore the effects of UVR on portal hypertension and liver reserve function (LRF). A total of 122 cirrhotic patients were enrolled and stratified into UVR and non-UVR groups based on the presence or absence of UVR. Clinical and laboratory indices, including hepatic biochemical parameters, portal venous system diameters, portal vein thrombosis, portal pressure gradient (PPG), Child-Pugh classification, and Model for End-Stage Liver Disease (MELD) scores, were compared between the 2 groups.

Compared with the non-UVR group, patients in the UVR group had significantly lower serum albumin levels (*P* = .032) and higher levels of aspartate aminotransferase (*P* = .040) and total bilirubin (*P* = .014). The UVR group also presented a markedly lower proportion of moderate-to-severe ascites (*P* = .032), whereas no significant intergroup difference in PPG was detected. Additionally, the UVR group exhibited a significantly higher proportion of Child-Pugh B and C grades (*P* = .031). Further subgroup analysis demonstrated that patients with a large UVR shunt (umbilical vein diameter > 6.48 mm) had worse Child-Pugh grade (*P* = .044) and higher MELD scores (*P* = .010) relative to non-UVR patients.

In conclusion, UVR may alleviate moderate-to-severe ascites by reducing superior mesenteric venous pressure. An umbilical vein diameter exceeding 6.48 mm may be a potential risk factor for deteriorated liver reserve function. Although UVR does not significantly decrease portal venous pressure, it may aggravate portal hemodynamic burden and facilitate the progression of liver cirrhosis.

## 1. Introduction

Liver cirrhosis ranks as the 11th leading global cause of mortality, with portal hypertension representing the primary fatal driver among cirrhotic patients.^[1,2]^ As a hallmark decompensation feature of cirrhosis, portal hypertension arises predominantly from elevated intrahepatic vascular resistance and subsequent venous congestion.^[3,4]^

The umbilical vein links the left portal vein branch to the periumbilical venous plexus and undergoes physiological obliteration following birth.^[5]^ In the setting of portal hypertension, extensive portocaval collateral vessels develop. In a subset of patients, recanalization of the obliterated umbilical vein establishes communications with thoracoabdominal veins, yielding the characteristic abdominal wall “jellyfish sign.” Theoretically, umbilical vein recanalization (UVR) could decompress the portal venous system, thereby lowering risks of esophageal variceal hemorrhage and ascites formation, and mitigating hypersplenism. Nevertheless, existing published data remain inconsistent regarding whether UVR effectively reduces portal hypertensive burden, as well as its influences on liver reserve function (LRF) and portal vein thrombosis (PVT).

Direct measurement of the portal pressure gradient (PPG) is technically cumbersome, so the hepatic venous pressure gradient (HVPG) is routinely adopted as a surrogate marker for PPG in clinical practice. Recent high-volume studies, however, have identified notable discrepancies between PPG and HVPG readings.^[6,7]^ To date, research investigating the correlation between UVR and PPG remains scarce. This study compared clinical profiles of cirrhotic patients undergoing transjugular intrahepatic portosystemic shunt (TIPS) stratified by the presence or absence of UVR. We further evaluated the impacts of UVR on portal hypertension severity, LRF, PPG, and PVT, aiming to generate clinical evidence and guide individualized management for cirrhotic patients complicated with UVR.

## 2. Materials and methods

### 2.1. Patient

**Figure 1.**
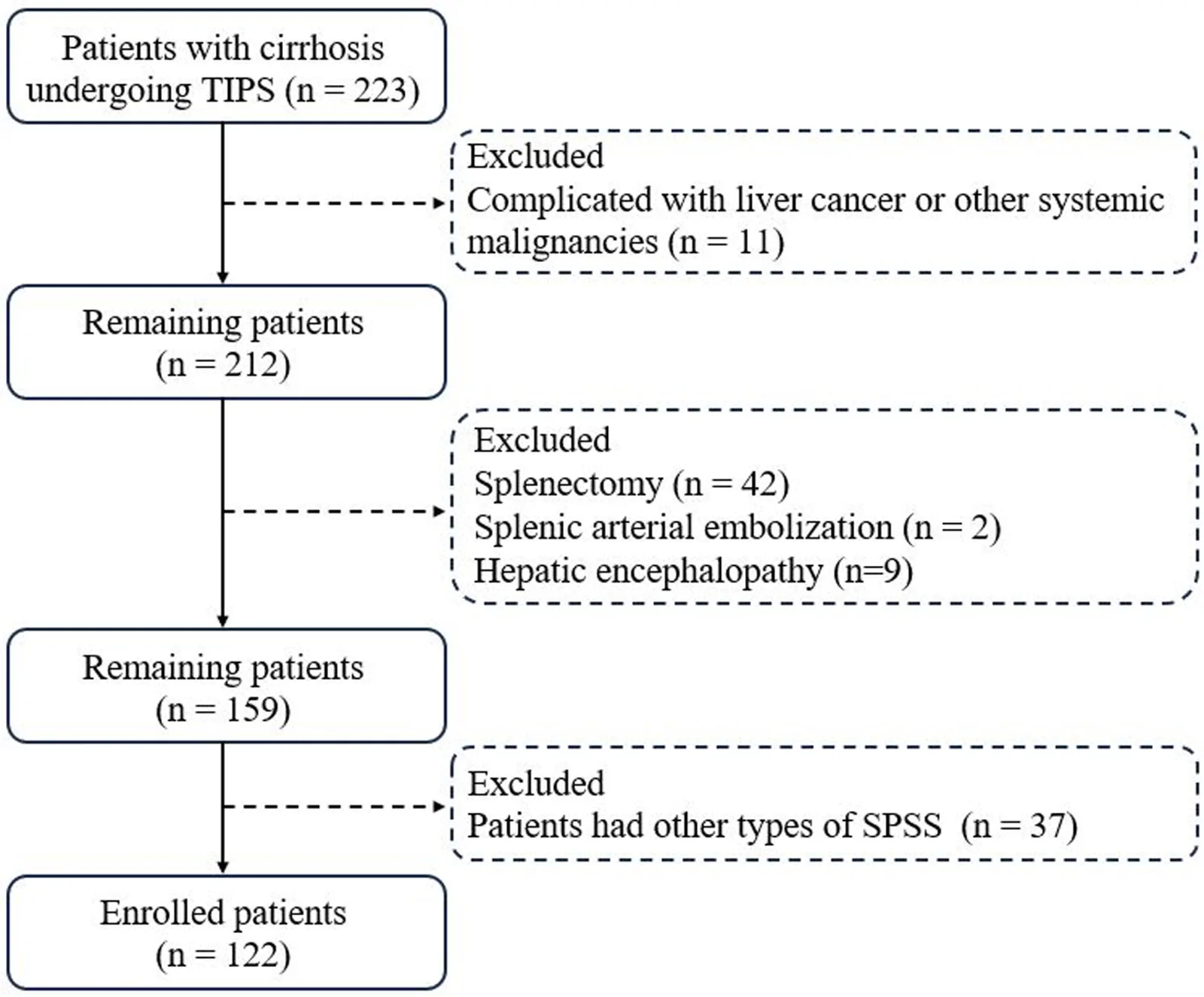
Flowchart of patients’ selection.

We retrospectively reviewed clinical data of cirrhotic patients who underwent transjugular intrahepatic portosystemic shunt (TIPS) between January 2017 and December 2023. The diagnosis of liver cirrhosis was confirmed based on clinical manifestations, physical examination, laboratory parameters, contrast-enhanced abdominal computed tomography (CT), magnetic resonance imaging (MRI), or liver histopathological findings, in accordance with the Chinese Guidelines for the Management of Liver Cirrhosis.^[8]^ All subjects received TIPS for recurrent esophagogastric variceal hemorrhage or refractory ascites. Patients satisfying any of the following criteria were excluded: concurrent hepatocellular carcinoma or other systemic malignancies; prior splenectomy, splenic artery embolization, or hepatectomy; preexisting splenorenal shunt, gastrorenal shunt, or ectopic varices; complicated hepatic encephalopathy; previous liver transplantation (Fig. 1). The present retrospective study obtained ethical approval from the Medical Ethics Committee of Zhongnan Hospital of Wuhan University.

### 2.2. Data collection procedure

**Figure 2.**
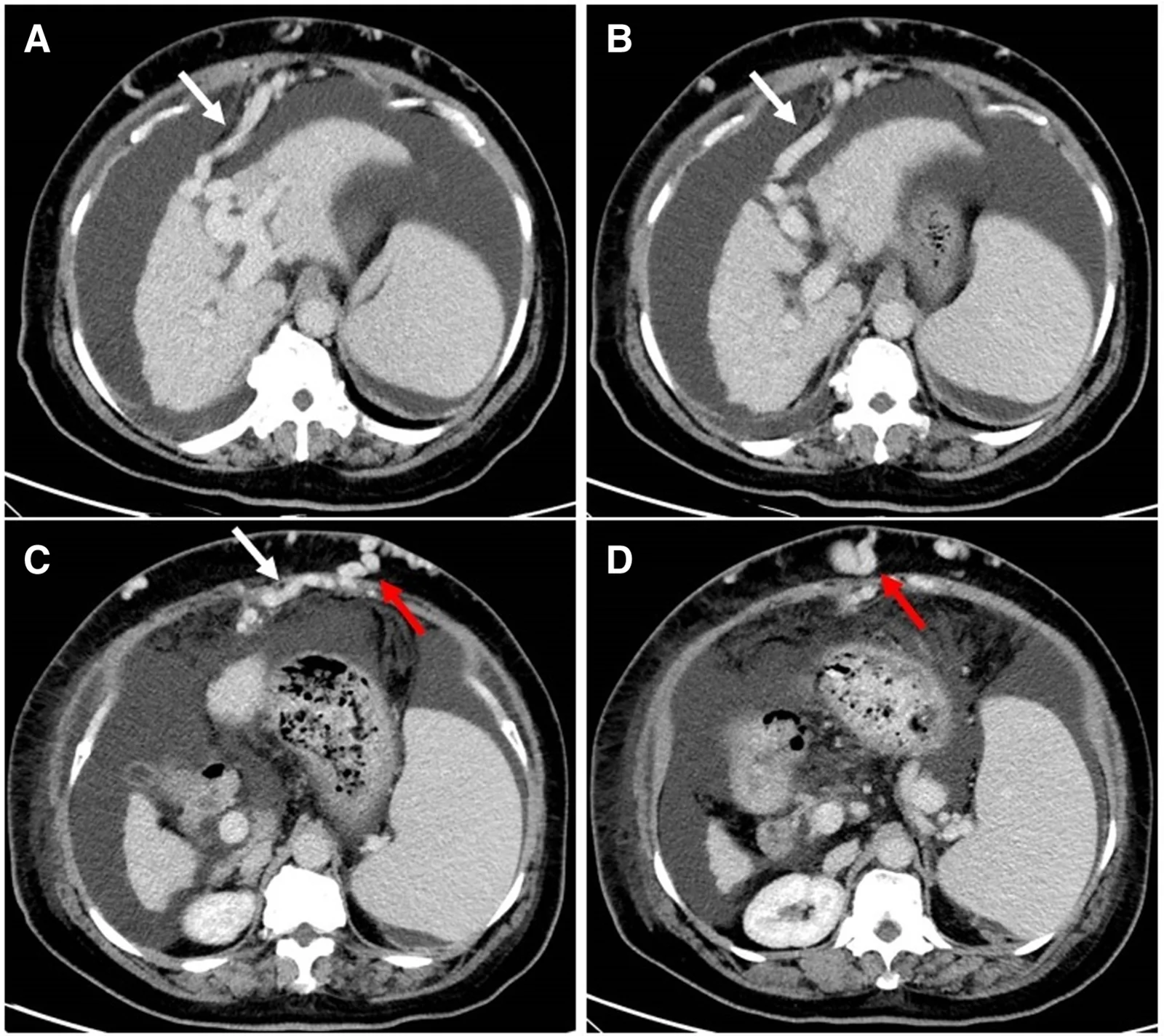
Abdominal contrast-enhanced CT showed umbilical vein recanalization with abdominal wall varicosis. The white arrow refers to umbilical vein recanalization with a diameter of approximately 7.3 mm. The red arrow refers to abdominal wall varicosis.

On contrast-enhanced CT and MRI, umbilical vein recanalization (UVR) manifests as a tubular collateral branch arising from the left portal vein and coursing toward the umbilicus, which can be clearly visualized during the portal venous phase of contrast enhancement (Fig. 2). Patients were stratified into a UVR group and a non-UVR group based on the presence or absence of UVR.

Baseline demographics and laboratory parameters were collected for all participants as follows: general characteristics: age, sex, underlying cirrhosis etiology, clinical symptoms, and physical signs; medical history: prior gastrointestinal hemorrhage episodes; laboratory biomarkers: red blood cell count (RBC), hemoglobin (HGB), white blood cell count (WBC), platelet count (PLT), prothrombin time (PT), international normalized ratio (INR), activated partial thromboplastin time (APTT), total bilirubin (TBIL), serum albumin (ALB), alanine aminotransferase (ALT), aspartate aminotransferase (AST), serum creatinine (CREA), blood urea nitrogen (BUN), and blood ammonia (Amon); scoring systems: Child–Pugh score and class, and Model for End-Stage Liver Disease (MELD) score. Any raw MELD value below 1 was standardized to 1 to avoid negative composite scores.

We measured the maximum luminal diameters of major portal venous system trunks, namely the main portal vein (MPV), left portal vein (LPV), right portal vein (RPV), superior mesenteric vein (SMV), and splenic vein (SV). Splenic longitudinal and anteroposterior thickness dimensions were also recorded. Splenic longitudinal diameter was defined as the maximum craniocaudal length measured at the splenic hilum midplane; splenic thickness represented the shortest transverse distance between the medial and lateral splenic margins at the same hilum level. Ascites was graded as mild (grade 1), moderate (grade 2), or severe (grade 3) in accordance with established clinical guidelines.^[9,10]^

### 2.3. Statistical analysis

All statistical analyses were conducted using SPSS 26.0 software. Continuous variables conforming to a normal or approximate normal distribution were presented as mean ± standard deviation and compared via the independent t-test. Non-normally distributed continuous variables were expressed as median (interquartile range, P25-P75) and analyzed using the Mann–Whitney U test. Categorical variables were assessed with the chi-square test, while Fisher’s exact test was applied when the minimum theoretical frequency was <5. Receiver operating characteristic (ROC) curve analysis was performed to determine the optimal cutoff value of umbilical vein diameter for predicting severe liver reserve dysfunction (MELD score > 10). A two-tailed *P* value < .05 was defined as statistically significant. Five patients had incomplete liver biochemistry data, which were handled via multiple imputation.

## 3. Results

### 3.1. General information in UVR and non-UVR patients

A total of 223 patients diagnosed with liver cirrhosis underwent TIPS during the study period. After strict screening according to the predefined exclusion criteria, 122 patients were ultimately enrolled in the present study (Fig. 1), among whom 39 had UVR and 83 did not, yielding an umbilical vein recanalization (UVR) incidence rate of 32.0%. Baseline demographic characteristics, including age (*P* = .242), sex distribution (*P* = .291), and cirrhosis etiology (*P* = .412), were comparable between the UVR and non-UVR groups (Table 1), with no significant intergroup differences observed.

**Table 1:** General information and etiology of cirrhosis

	Non-UVR (N = 83)	UVR (N = 39)	*P*
Age (years) (Mean ± SD)	58.1 ± 10.3	55.6 ± 11.7	0.242
Gander, n (%)			
Male	47 (56.6%)	26 (66.7%)	0.291
Female	36 (43.4%)	13 (33.3%)	
Pathogenesis, n (%)			
Viral hepatitis	52 (62.7%)	25 (64.1%)	0.412
Alcohol	5 (6.0%)	3 (7.7%)	
Nonalcoholic fatty liver	5 (6.0%)	0 (0.0%)	
Autoimmune	7 (8.4%)	6 (15.4%)	
Others	14 (16.9%)	5 (12.8%)	

UVR, umbilical vein recanalization.

Others: schistosomiasis, Wilson’s disease, drugs, cryptogenic, etc.

### 3.2. Comparison of laboratory test results

Compared with the non-UVR group, patients in the UVR group exhibited significantly lower serum albumin (ALB) levels (27.6 ± 5.1 g/L vs 29.8 ± 5.3 g/L, *P* = .032) and significantly higher total bilirubin (TBIL) levels (24.9 μmol/L vs 20.6 μmol/L, *P* = .014) and aspartate aminotransferase (AST) levels (*P* = .040). No significant differences were observed between the 2 groups in other laboratory indices, including RBC (*P* = .691), HGB (*P* = .575), WBC (*P* = .469), PLT (*P* = .845), PT (*P* = .312), INR (*P* = .310), APTT (*P* = .519), ALT (*P* = .066), CREA (*P* = .143), BUN (*P* = .289), and AMON (*P* = .791) (Table 2).

**Table 2:** Comparison of laboratory test results between UVR group and non-UVR group

	Non-UVR (N = 83)	UVR (N = 39)	*P*
RBC (10^12^/L)	2.48 ± 0.56	2.53 ± 0.70	0.691
HGB (g/L)	70.3 ± 20.1	72.6 ± 22.3	0.575
WBC (10^9^/L)	3.5 (2.25–5.00)	3.4 (2.4–7.39)	0.469
PLT (10^9^/L)	66 (50–85)	68 (40–84)	0.845
PT (s)	15.1 (14.0–17.6)	16.3 (14.2–18.5)	0.312
INR	1.38 (1.28–1.61)	1.49 (1.30–1.69)	0.310
APTT (s)	31.4 ± 3.99	31.9 ± 3.77	0.519
TBIL (μmol/L)	20.6 (14.6–27.7)	24.9 (18.7–37.4)	0.014
ALB (g/L)	29.8 ± 5.3	27.6 ± 5.1	0.032
ALT (U/L)	20 (14–29)	24 (17–41)	0.066
AST (U/L)	27 (22–43)	44 (25–53)	0.040
CREA (μmol/L)	67.1 (57.1–79.0)	72.5 (64.2–86.2)	0.143
BUN (mmol/L)	6.97 (4.51–9.60)	6.50 (5.51–10.2)	0.289
AMON (μmol/L)	44.84 (33.83–58.38)	46.15 (29.77–66.32)	0.791

ALB = albumin, ALT = alanine aminotransferase, AMON = ammonia, APTT = activated partial prothrombin time, AST = aspartate aminotransferase, BUN = blood urea nitrogen, CREA = creatinine, HGB = hemoglobin, INR = international standardized ratio, PLT = platelet count, PT = prothrombin time, RBC = red blood cell count, TBIL = total bilirubin, UVR = umbilical vein recanalization, WBC = white blood cell count.

### 3.3. Comparison of the incidence of portal vein thrombosis (PVT)

Similarly, the 2 groups showed comparable rates of portal vein thrombosis (33.3% vs 38.6%; *P* = .577) and portal vein cavernous transformation (0.0% vs 2.4%; *P* = 1.000) (Table 3), with no statistically significant differences detected.

**Table 3:** The incidence of PVT and PVS between UVR group and non-UVR group

	Non-UVR (N = 83)	UVR (N = 39)	*P*
PVT	32 (38.6%)	13 (33.3%)	0.577
Non-PVT	51 (61.4%)	26 (66.7%)	
PVS	2 (2.4%)	0 (0.0%)	0.831
Non-PVS	81 (97.6%)	39 (100%)	

PVS = portal vein spongiosis, PVT = portal vein thrombosis, UVR = umbilical vein recanalization.

### 3.4. Clinical manifestations of portal hypertension

The major clinical manifestations of portal hypertension consist of ascites, splenomegaly, and collateral vessel recanalization. The overall incidence of ascites was comparable between the UVR and non-UVR groups (76.9% vs 74.7%, *P* = .790). Subgroup analysis of ascites severity revealed that the UVR group had rates of 23.1% for no ascites, 41.0% for mild ascites, and 35.9% for moderate-to-severe ascites, whereas the non-UVR group had corresponding rates of 25.3%, 19.3%, and 55.4%. Notably, the UVR group presented with a significantly lower proportion of moderate-to-severe ascites than the non-UVR group (*P* = .032) (Table 4).

**Table 4:** Comparison of clinical manifestations of portal hypertension between UVR group and non-UVR group

	Non-UVR (N = 83)	UVR (N = 39)	*P*
Ascites	62 (74.7%)	30 (76.9%)	0.790
None	21 (25.3%)	9 (23.1%)	0.032
Mild	16 (19.3%)	16 (41.0%)	
Moderate-severe	46 (55.4%)	14 (35.9%)	
MPV (mm)	16.26 ± 2.42	16.88 ± 2.56	0.206
LPV (mm)	11.75 ± 2.26	12.24 ± 1.91	0.240
RPV (mm)	11.39 ± 1.86	11.57 ± 2.54	0.664
SV (mm)	12.33 ± 2.30	13.20 ± 3.16	0.128
SMV (mm)	13.44 ± 2.15	13.08 ± 2.63	0.421

UVR, umbilical vein recanalization; MPVm main portal vein; LPV, left portal vein; RPV, right portal vein; SMV, superior mesenteric vein; SV, splenic vein.

Patients in the UVR group exhibited significantly larger splenic dimensions than those in the non-UVR group, including longer splenic length diameter (14.94 ± 2.58 cm vs 13.80 ± 1.94 cm, *P* = .008) and greater splenic thickness diameter (7.67 ± 1.37 cm vs 7.12 ± 1.40 cm, *P* = .045).

In the UVR group, the median umbilical vein diameter was 5.63 mm (range, 3.03–13.34 mm). No significant intergroup differences were observed in the diameters of major portal venous system vessels, including the MPV (*P* = .206), LPV (*P* = .240), RPV (*P* = .664), SV (*P* = .128), and SMV (*P* = .421) (Table 4). Additionally, the internal diameter of the gastric coronary vein was similar between the 2 groups (7.10 ± 2.43 mm vs 7.75 ± 2.69 mm, *P* = .202).

### 3.5. Comparison of liver reserve function

The UVR group exhibited a higher median Child-Pugh score than the non-UVR group (9 vs 8), though the difference did not reach statistical significance (*P* = .188). In terms of Child-Pugh classification, the UVR group comprised 3 cases of grade A, 22 cases of grade B, and 14 cases of grade C, while the non-UVR group included 20 grade A, 38 grade B, and 25 grade C cases. The overall distribution of Child-Pugh grades was similar between the 2 groups (*P* = .097). However, the UVR group had a significantly higher proportion of combined Child-Pugh B and C grades (*P* = .031) (Table 5). Additionally, the UVR group showed numerically higher MELD scores than the non-UVR group [12.75 (9.17–15.09) vs 10.89 (9.40–14.15)], with no significant intergroup difference (*P* = .293) (Table 5).

**Table 5:** Comparison of LRF between UVR group and non-UVR group

	Non-UVR (N = 83)	UVR (N = 39)	P	large shunt subgroup (N = 16)	P	small shunt subgroup (N = 23)	P
MELD score	10.89	12.75	0.293	15.09	0.004	12.41	0.393
Child-Pugh							
A	20 (24.1%)	3 (7.7%)	0.097	0 (0)	0.038	3 (13.0%)	0.244
B	38 (45.8%)	22 (56.4%)		7 (43.8%)		15 (65.2%)	
C	25 (30.1%)	14 (35.9%)		9 (56.3%)		5 (21.7%)	
A	20 (24.1%)	3 (7.7%)	0.031				
B + C	63 (75.9%)	36 (92.3%)					

LFR = liver reserve function, MELD = model for end-stage liver disease, UVR = umbilical vein recanalization.

ROC curve analysis was performed to determine the optimal cutoff value of umbilical vein diameter for predicting severe liver reserve dysfunction, defined as a MELD score > 10. The optimal cutoff diameter was 6.48 mm, with an area under the curve of 0.800 (*P* = .002). Based on the 6.48 mm cutoff value, patients with UVR were further stratified into a small shunt subgroup (≤6.48 mm) and a large shunt subgroup (>6.48 mm). The small shunt subgroup consisted of 3 grade A, 15 grade B, and 5 grade C cases, with no significant difference in Child-Pugh grade distribution compared with the non-UVR group (*P* = .244). In contrast, the large shunt subgroup included no grade A cases, 7 grade B cases, and 9 grade C cases, demonstrating a significantly different Child-Pugh grade profile relative to the non-UVR group (*P* = .038). Although the large shunt subgroup had a higher proportion of Child-Pugh B and C grades than the small shunt subgroup, the difference was not statistically significant (*P* = .255).

For MELD scores, the small shunt subgroup showed slightly lower values than the non-UVR group [12.41 (9.17–14.31) vs 10.89 (9.40–14.15)], without significant difference (*P* = .393). Notably, the large shunt subgroup yielded significantly higher MELD scores than both the non-UVR group [15.09 (13.85–16.41) vs 10.89 (9.40–14.15), *P* = .004] and the small shunt subgroup [15.09 (13.85–16.41) vs 12.41 (9.17–14.31), *P* = .009] (Table 5).

### 3.6. Comparison of portal pressure gradient

All enrolled patients in both the UVR and non-UVR groups received TIPS for recurrent esophagogastric variceal bleeding or refractory ascites. The UVR group exhibited a numerically higher portal pressure gradient (PPG) than the non-UVR group (24.77 ± 4.84 mm Hg vs 23.50 ± 4.45 mm Hg), though the difference did not reach statistical significance (*P* = .158). Subgroup analysis showed that both the large shunt subgroup (24.46 ± 5.26 mm Hg vs 23.50 ± 4.45 mm Hg, *P* = .447) and the small shunt subgroup (24.98 ± 4.63 mm Hg vs 23.50 ± 4.45 mm Hg, *P* = .165) had slightly elevated PPG relative to the non-UVR group, with no significant intergroup differences. In addition, no significant difference in PPG was observed between the large and small shunt subgroups (24.46 ± 5.26 mm Hg vs 24.98 ± 4.63 mm Hg, *P* = .745).

## 4. Discussion

Portal hypertension is a predominant complication of liver cirrhosis, primarily driven by increased hepatic vascular resistance and portal venous stasis. A portal venous pressure exceeding 10 mm Hg triggers the formation of extensive portosystemic collateral circulations, including umbilical vein recanalization (UVR), gastrorenal shunt, splenorenal shunt, rectal varices, and retroperitoneal varices.^[11–13]^ As the only portal venous collateral aligned with hepatic blood outflow, UVR features higher portal perfusion and lower vascular resistance compared with other collateral pathways.^[14]^ To date, the impacts of UVR on portal venous pressure and liver reserve function (LRF) remain controversial.

The reported prevalence of UVR varies widely from 7.4% to 82%, with a higher incidence in patients with alcoholic cirrhosis than in those with viral hepatitis-related cirrhosis.^[15–18]^ In the present study, the overall UVR rate was 32%. No significant correlation was observed between UVR prevalence and cirrhosis etiology, and the proportion of alcoholic cirrhosis was comparable between UVR and non-UVR groups.

Portal vein thrombosis (PVT), with a cirrhosis-associated incidence of 0.6–15.8%,^[19]^markedly elevates the risks of gastrointestinal bleeding, hepatorenal syndrome, refractory ascites, and posttransplant mortality, and is closely correlated with progressive liver dysfunction.^[20–22]^ PVT development is attributed to hypercoagulability, portal hemodynamic disorders, and vascular endothelial injury. A previous study demonstrated that UVR does not increase PVT risk but reduces the incidence of portal vein cavernous transformation.^[23]^ Consistent with these findings, our study showed no significant differences in PVT and portal cavernous transformation rates between the 2 groups, indicating that UVR is not associated with altered blood coagulation status or substantial portal hemodynamic disruption.

Prior studies have reported no significant changes in serum albumin (ALB), prothrombin time (PT), and bilirubin levels in UVR patients, suggesting preserved hepatic synthetic and biliary excretory function following UVR.^[23]^ However, our results revealed significantly lower ALB levels and elevated aspartate aminotransferase (AST) and total bilirubin (TBIL) levels in the UVR group, implying potential impairments in hepatic albumin synthesis, hepatocyte integrity, and bilirubin metabolism in UVR patients. The discrepancy between studies may stem from inconsistent inclusion criteria: previous studies enrolled general liver disease patients, whereas the current study exclusively included cirrhotic patients with TIPS indications. We hypothesize that UVR may exacerbate the adverse effects of gastrointestinal bleeding on hepatic perfusion, thereby compromising hepatic synthetic and metabolic function. Additionally, unstandardized preoperative albumin supplementation across participating hospitals may have confounded the results; such confounding factors could not be fully adjusted for due to the retrospective nature of this study.

Ascites, splenomegaly, and collateral circulation formation are hallmark clinical manifestations of portal hypertension. Theoretically, UVR alleviates portal hypertension by diverting portal venous blood into the systemic circulation. In this cohort, the overall incidence of ascites was similar between groups, yet the UVR group presented a significantly lower proportion of moderate-to-severe ascites and smaller superior mesenteric vein (SMV) diameter, suggesting that UVR may mitigate ascites severity by reducing SMV pressure. Although the main portal vein, left portal vein, right portal vein, and splenic vein diameters were slightly higher in the UVR group, the differences were not statistically significant. Notably, the UVR group exhibited significantly larger splenic length and thickness. Direct pressure measurement further confirmed higher portal pressure gradient (PPG) in UVR patients. Collectively, these findings indicate that UVR cannot relieve elevated pressure in the main portal vein and splenic vein caused by portal hypertension but can improve ascites via reducing SMV pressure.

Umbilical vein shunting diverts intrahepatic portal blood flow, which may theoretically impair liver function. Existing studies on UVR-related liver reserve changes remain inconsistent: one study reported improved Child-Pugh grades but unchanged Model for End-Stage Liver Disease (MELD) scores after UVR, while another found a higher proportion of Child-Pugh grade C in UVR patients.^[23,24]^ Our study detected numerically higher MELD and Child-Pugh scores in the UVR group without statistical significance. Subgroup analysis further demonstrated that patients with large UVR shunts (diameter > 6.48 mm) had significantly higher MELD scores and a higher proportion of Child-Pugh B/C grades than non-UVR patients, whereas small shunts (≤ 6.48 mm) showed no significant impacts on these indicators. Moreover, the large-shunt subgroup had worse liver function scores than the small-shunt subgroup. These results indicate that small-caliber UVR has minimal effects on LRF, while UVR shunts exceeding 6.48 mm are an independent risk factor for deteriorated hepatic reserve. It is speculated that embolization of large-diameter (> 6.48 mm) patent umbilical veins may improve liver function but could exacerbate ascites; further prospective studies are required to validate this conclusion. This cutoff was derived from ROC analysis in our cohort and requires validation in independent studies before clinical application.

The association between UVR and variceal bleeding risk remains debated. Some scholars proposed that UVR mimics the hemodynamic effect of TIPS to reduce portal pressure and prevent esophagogastric variceal rupture, with clinical studies observing a higher UVR prevalence in patients with mild esophageal varices and speculating that large-caliber UVR (> 6.48 mm) reduces bleeding risk.^[25,26]^ Conversely, other studies challenged this protective effect, reporting that UVR correlates with severe liver damage and advanced esophageal varices, with elevated hepatic venous pressure gradient (HVPG) in UVR patients.^[18,27]^ In the present study, most enrolled patients had recurrent esophagogastric variceal bleeding, limiting the direct evaluation of UVR on bleeding risk. Theoretically, UVR may reduce portal blood perfusion to gastric coronary veins and alleviate varices. However, no significant reduction in gastric coronary vein diameter was observed in the UVR group, indicating that UVR fails to reduce coronary venous portal inflow and cannot mitigate esophagogastric varices or lower bleeding risk.

UVR and spontaneous splenorenal shunt (SSRS) are the 2 most common spontaneous portosystemic shunts. Consistent with SSRS, UVR is associated with reduced serum albumin and impaired liver function, corresponding to higher Child-Pugh and MELD scores.^[28,29]^ However, UVR differs from SSRS in that SSRS increases PVT risk and reduces portal pressure, whereas UVR exerts no significant effects on PVT occurrence and portal pressure reduction.

This study has several limitations. First, the restrictive inclusion criteria precluded the evaluation of UVR’s impacts on other cirrhosis-related complications, including hepatic encephalopathy, variceal bleeding, and hepatocellular carcinoma. Second, this is a single-center retrospective study with a relatively small UVR sample size (n = 39), which may introduce statistical bias. Future large-sample, multicenter studies are needed to verify our findings. Third, preoperative medications (e.g., entecavir, tenofovir, diuretics, ursodeoxycholic acid, propranolol) may have interfered with liver function and hemodynamic outcomes. Fourth, multiple unmeasured confounding factors, including hypertension history, long-term medication use, and endoscopic treatment frequency, may have affected the results. Well-designed prospective studies are warranted to eliminate these confounding effects and clarify the exact clinical role of UVR in cirrhotic patients undergoing TIPS. Fifth, to investigate the effect of UVR on portal venous pressure, only cirrhotic patients with UVR who underwent TIPS were enrolled in this study, which may lead to data bias.

## 5. Conclusion

Umbilical vein recanalization (UVR) reflects elevated portal hemodynamic burden and serves as an indicator of progressive cirrhosis. First, UVR impairs hepatic synthetic and excretory function, manifesting as compromised albumin synthesis and impaired bilirubin metabolism. Second, while UVR fails to decompress the portal and splenic veins, it alleviates ascites by lowering superior mesenteric venous pressure. Third, UVR does not mitigate the risk of esophageal and gastric variceal hemorrhage. Fourth, an umbilical vein diameter exceeding 6.48 mm may be an independent risk factor for worsening liver reserve function (LRF).

## Acknowledgements

Liping Chen and Jing Wang are co-corresponding authors for this manuscript.

## Author contributions

**Data curation:** Feng Ding.

**Methodology:** Feng Ding.

**Writing – original draft:** Feng Ding.

**Conceptualization:** Liping Chen.

**Project administration:** Liping Chen, Jing Wang.

**Software:** Yuan Yang.

**Writing – review & editing:** Yuan Yang, Jing Wang.
